# Co-occurrence Strength and Transitivity Effects on Spanish Clitic Case Variation With Reverse-Psychological Predicates

**DOI:** 10.3389/fpsyg.2021.712959

**Published:** 2021-07-19

**Authors:** Gustavo Guajardo

**Affiliations:** Department of Language and Culture, AqcVA Aurora Research Centre, UiT, The Arctic University of Norway, Tromsø, Norway

**Keywords:** association measure, co-occurrence, clitic, psychological predicates, Log Dice, Spanish

## Abstract

Although the most frequent psychological predicates in Spanish require the third-person clitic experiencer to appear in dative case, there is a well-known subclass of predicates for which the case of the clitic alternates between accusative and dative. This alternation has been previously accounted for by certain grammatical properties of the clause containing the clitic as well as elements of transitivity. However, since most studies on the subject have only looked at a subset of the elements comprising transitivity, it remains to be demonstrated whether the alternation in clitic case can reliably be reduced to a difference in transitivity. In this paper, I study the extent to which transitivity is the main predictor of clitic case alternation with reverse psychological predicates by comparing its effect with another potential predictor, namely the bidirectional association strength between the verb and the clitic. The results show that higher levels of association between the clitic and the verb favor the dative clitic, suggesting a higher degree of lexicalization of the dative clitic-verb pair. Furthermore, although it is found that higher levels of transitivity favor the accusative clitic, the effect is rather small compared to the rest of the predictors. All in all, the results support previous findings in the literature, but they also bring to the fore the importance of frequency of co-occurrence on Spanish clitic case alternation in particular, and language variation more generally.

## Introduction

The morphosyntactic mapping of clitic case in Spanish is far from being uniform and there are several cases in which the syntactic function of the clitic does not match its morphological form. Perhaps the best-known example of this disparity between function and form is the phenomenon referred to as *leísmo* (Bello, 1898; Roldán, 1975), in which the morphologically third-person dative clitic is used with direct objects that are animate and masculine.

But there are other examples where clitic case and syntactic function do not align. For example, with causative predicates such as *hacer* and *dejar* the third person clitic can alternate between accusative and dative with no apparent or clear change in meaning. This case alternation has recently been accounted for by resorting to the concept of clausal transitivity (Hopper and Thompson, 1980). Guajardo (2021) shows that higher levels of transitivity favor the dative clitic, a preference driven by the tendency of transitive verbs to prefer the dative clitic.

This paper examines a subset of so-called reverse-psychological (henceforth r-psych) predicates which are another well-known context in which the case of the clitic can alternate between accusative and dative (1–2)^1^. The reverse part of the name refers to the fact that the experiencer in this type of predicate is not expressed in the more canonical nominative form.

1. […] **le aterra** la idea de que elija a su familia biológica.
  “[…] the idea that s/he should choose her/his biological family terrifies her(DAT)”
  (Panama: 2943)
2. […] **la aterra** la libertad.
  “[…] freedom terrifies her(ACC)” (Chile: 617)

In (1–2), the r-psych predicate *aterrar* “to terrify” appears in both examples but in (1) the experiencer is realized in the dative case while in (2) it appears in accusative. These two examples highlight the probabilistic nature of the phenomenon. Note that the two sentences share the same verb *aterrar* “to terrify” in the present tense and the subject is inanimate and feminine in both, yet they differ in the case marking of the clitic.

This phenomenon has been studied from several different approaches ranging from purely theoretical accounts (Parodi and Luján, 2000; Ackerman and Moore, 2001; Cuervo, 2003; Fábregas and Marín, 2020) to more functionalist and typological approaches, many of which are based on Hopper and Thompson (1980)’s concept of transitivity (Vázquez Rosas, 2006; Harris et al., 2011; Miglio et al., 2013; Ganeshan, 2015). Most studies that have used transitivity as the main explanation of the alternation have mostly focused on a subset of the transitivity parameters as well as other grammatical features of the clause such as grammatical aspect, whether the subject is clausal or lexical, and genre. A potential problem with these analyses is the arbitrary choice of a subset of the transitivity parameters, which is then used to claim that the difference in clitic case can be accounted for by transitivity. An additional shortcoming of some previous analyses is the relatively small datasets or the lack of statistical analysis in others. Clitic case alternation in Spanish is clearly a phenomenon that is probabilistic in nature and not categorical (Vázquez Rosas, 2006). Therefore, any analysis that is based on the premise of grammatical categorical distinctions is bound to be limited in scope and will fall short in providing a satisfactory account of the phenomenon.

In order to address these issues, the account presented herein rests on three main pillars: (i) a relatively large dataset of over 4,000 sentences, (ii) using all transitivity parameters in the form of the Transitivity Index (Guajardo, 2021) and (iii) a mixed-effects logistic regression model to study the effects of all predictors on clitic case alternation.

In addition, I introduce a new type of predictor whose effect has not been studied before. This predictor is a measure of the association strength between the clitic and the verb. As is well-known in the corpus linguistics literature, there are many different ways to calculate the association strength between two or more words, all of which have their strengths and weaknesses focusing on different properties of the association. In the present work, I use the Log Dice (Rychlý, 2008), which measures the exclusive association of two words in a corpus (Gablasova et al., 2017). The motivation for such a variable is to assess whether certain *clitic+verb* combinations are more likely than others, which may weaken the explanatory power of transitivity in the clitic case alternation. However, it is also possible that by adding this variable into the statistical model, the role of transitivity may be boosted up because both variables may explain different aspects of the alternation. This is the main question I seek to answer in the present work.

The results show that when transitivity is computed as a global measure of the clause its effect is quite small and subject to regional variation. In contrast, the association strength between the clitic and the verb is a much stronger predictor of the alternation, with higher levels of association favoring the dative clitic but with a higher degree of regional variation than transitivity. In addition, in line with previous analyses, I show that clausal subjects and non-perfective verb forms favor the dative clitic as do subjects in the 3rd person.

## Transitivity

In their seminal paper, Hopper and Thompson (1980) propose that transitivity should be understood as a property of the whole clause and not just the verb. They decompose the notion of transitivity into 10 different parameters, shown in Table 1. The parameters are all binary, whose values can be either High or Low (in transitivity). For example, for the PARTICIPANTS parameter, a transitive verb would score High whereas an intransitive verb would receive Low. Crucial to their proposal is the idea that transitivity is the summation of the values of all parameters such that no single value can determine whether a clause is high or low in transitivity. They illustrate this idea by showing that an intransitive clause may be higher in transitivity than a transitive clause if other elements of transitivity score high in the intransitive clause. This can be achieved, for example, if the subject of the intransitive verb is an agent or highly individuated (e.g., definite, specific, singular, animate) while the subject of the transitive verb is not.

**TABLE 1:** Transitivity parameters from Hopper and Thompson (1980).

Parameter	Low	High	Parameter	Low	High
PARTICIPANT	1	2 or more	AFFIRMATION	Non-affirmative	Affirmative
KINESIS	Stative	Action	MODE	Irrealis	Realis
ASPECT	Atelic	Telic	AGENCY	A low in agency	A high in agency
PUNCTUALITY	Non-punctual	Punctual	AFFECTEDNESS O	Not affected	Totally affected
VOLITIONALITY	Non-volitional	Volitional	INDIVIDUATION O	Non-individuated	Highly individuated

The parameter individuation deserves some clarification as this is best understood as a super-parameter made up of six subparameters referring to the object. These subparameters are (i) proper vs. common noun, (ii) human/animate vs. inanimate, (iii) concrete vs. abstract, (iv) singular vs. plural, (v) count vs. mass, and (vi) referential/definite vs. non-referential. The values of each subparameter refer to high and low levels of transitivity, respectively.

A criticism of this proposal that has often been pointed out is the lack of hierarchy among the parameters (Givón, 1985; Malchukov, 2006). In other words, the original proposal assumes that all parameters are equally important regardless of the construction. There are clear reasons why this may be problematic. For example, it is not difficult to imagine that alternations in the noun phrase (NP) are probably more sensitive to those parameters concerned with features of the noun than to those of the verb. For example, in differential object marking (DOM), whether or not the object gets marked depends on features of the NP object such as animacy, definiteness or specificity depending on the language. These parameters are likely to play a more decisive role in DOM than, say, features of the verb. However, this does not amount to saying that when studying DOM we should only investigate features of the object and ignore the rest of the parameters. What it means is that the importance of the parameters cannot be the same across constructions and the contribution of each of the transitivity parameters is likely to fluctuate construction by construction. The importance of each parameter in different constructions is far from obvious, however, and should therefore be determined empirically. The implementation of transitivity in the present article addresses this issue directly by using the Transitivity Index (Guajardo, 2021), which is a weighted measure of transitivity. The index is calculated in a way that the relevance (technically, the weight) of each parameter in the specific construction under study is taken into consideration. As a result, the hierarchical ordering of the parameters changes in a dynamic fashion, reflecting the particular characteristics of the construction being investigated (see section “Materials and Methods” for more details).

In the present article, the calculation of the index from Guajardo (2021) has been adapted slightly following Vázquez Rosas (2006)’s suggestion that the INDIVIDUATION parameter should also be included for the subject and not just the object. Therefore, INDIVIDUATION A was included to the list of the original parameters resulting in a total of 11 parameters.

With this theoretical framework in place, I will now discuss previous analyses that have implemented the notion of transitivity to account for the clitic-case alternation with reverse-psychological predicates.

### Vázquez Rosas (2006)

Vázquez Rosas (2006) examines two classes of psychological predicates, namely those that only take a dative clitic and those that can alternate between accusative and dative. Her analysis is based on corpus data from *Base de Datos Sintácticos* “Syntactic Data Base,” which is a corpus that includes all the texts in the Archive of Hispanic Texts of the University of Santiago.

For the class of r-psych verbs that only allows the dative clitic, she argues that these predicates are characterized by low transitivity because they appear in clauses that are stative, atelic and non-punctual. Although she finds examples in her data to back up her claims, she acknowledges that it is “impossible to establish a direct relationship between syntactic pattern and the stative or dynamic nature of the situation denoted by the verb” (Vázquez Rosas, 2006, p. 88).

With respect to the predicates that allow the clitic case alternation, she finds that accusative marking correlates with dynamic and telic clauses, animate subjects and affected objects. In contrast, those with a dative clitic tend to appear with stative and atelic clauses, inanimate subjects and objects that are psychologically affected. Based on these observations, she concludes that accusative marking signals high transitivity whereas dative marking shows low transitivity.

As acknowledged by Vázquez Rosas(2006, p. 107), the conclusions presented in her work must be taken as preliminary, however, given the small data sample used in the study of the verbs that can alternate the clitic case, with a total of just 154 sentences, of which only 21 had a third-person clitic.

### Miglio et al. (2013)

The point of departure of Miglio et al. (2013)’s work is Vázquez Rosas (2006) and Harris et al. (2011), the latter being a conference paper by one of the authors. Their study is based on corpus data from Corpus del Español (Davies, 2002), where they extract 55 verbs identified by Vázquez Rosas (2006) as having an experiencer that can alternate between accusative and dative. Their analysis is based on a dataset of 1,656 clauses. The contribution of their study is that they include genre as part of the possible predictors and they analyse the data with a mixed-effects logistic regression model, thus allowing them to conduct a statistical assessment of the tendencies in the data.

The predictor variables they study are animacy of the stimulus, whether the stimulus was clausal or not, tense (imperfect vs. present vs. perfect vs. preterit), mood and genre (academic, vs. literature vs. news vs. oral). They also included random effects of author and verb.

The most important effect they find is the animacy of the subject, such that inanimate subjects favor dative marking. In addition, they find two interactions. One interaction between genre and tense and a second interaction between genre and clausal stimulus. The most important finding with respect to tense is that the two more atelic tenses (imperfect and present) behave in a similar fashion in that they both have a low probability of the dative clitic whereas news and oral data show a high probability of this clitic. With respect to the more telic tenses (preterite and perfect) the picture is less straightforward. There appears to be a preference for accusative in the literature genre but dative in oral data. However, academic writing is found with accusative with perfect but oblique with preterite. The second interaction between genre and clausal stimulus shows that all genres except academic writing prefer dative marking with clausal subjects.

**FIGURE 1:**
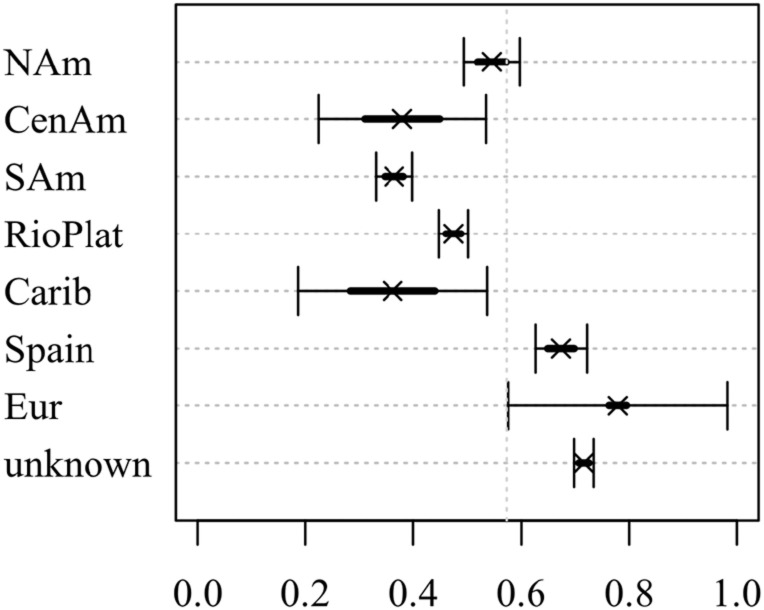
Predicted probabilities of dative clitic from Miglio et al.(2013: 275).

They further claim that there appears to be a geographical cline in the use of clitic case in this construction. They group the authors in their dataset into eight separate geographical regions: North America, Central America, South America, Rio de la Plata, Caribbean, Spain, Europe and unknown^2^. According to the authors, the use of accusative marking seems to increase as one goes down from North America to South America. However, their results, shown in Figure 1, seem to support an analysis where the distinction is between North American Spanish and the rest of Latin America with no clear gradual cline as they suggest.

The conclusion of the authors is that the clitic case alternation can be predicted from semantic factors related to transitivity, namely animacy and agentivity (Miglio et al., 2013, p. 276–277). However, since they do not code the data for agentivity, it is not clear how they reach this conclusion as animacy and agentivity are two separate, independent properties. Also, since they only analyse animacy of the subject, the claim that transitivity is involved in the alternation seems premature. Transitivity is made up of 10 parameters so the behavior of only one of them can hardly be used to support a claim about the effect of transitivity as a whole.

A confounding factor in their data is the fact that Peninsular Spanish is included in the study. The problem with this is that Peninsular Spanish uses the morphologically dative clitic to mark masculine animate/human objects, a phenomenon known in the literature as *leísmo* (e.g., Landa, 1995; Fernández-Ordoñez, 1999; Bleam, 2000; Ormazabal and Romero, 2013). Thus, use of a morphologically dative clitic in this variety does not entail dative marking, rendering it impossible to determine the actual grammatical case the speaker intended^3^.

### Ganeshan (2015)

Ganeshan (2015) takes a lexical semantic approach based on transitivity. She uses corpus data and native speaker judgments in her analysis and claims that the clitic case alternation with reverse psychological predicates in Spanish can be accounted for by resorting to two of the transitivity parameters, namely agentivity and affectedness of the object. More specifically, she claims that accusative marking is unspecified for agentivity and entails affectedness of the object, while dative case entails a weakening or lack of agentivity and is unspecified for affectedness of the object. She further suggests that her findings are predicted by Hopper and Thomposon’s (1980) Transitivity Hypothesis in that “in two reverse psychological verb clauses that differ, the features agentivity, affectedness, and case marking co-vary in the same direction” (Ganeshan, 2015, p. xii).

An important claim by Ganeshan is the difference between animacy and agentivity. She claims that although animacy can act as a proxy for agentivity (as in Miglio et al., 2013), the key parameter is really agentivity because an inanimate stimulus may appear with an accusative clitic. This conclusion is, however, not really tested in her work because in order to do this, one should compare animacy with agentivity and see which of the two parameters is a better predictor of clitic case. Needless to say, the fact that one can find examples of inanimate stimuli with the accusative clitic does not warrant the conclusion that animacy is not relevant and therefore agency must be.

Perhaps one of the most important contributions of her work is that she is explicit about how she determines the value of the transitivity parameters (e.g., affectedness, agentivity, volitionality, kinesis). She adapts specific tests from other authors to Spanish and therefore this facilitates replicability. Thus, in the present study I adopted her tests to determine the values of the transitivity parameters. The tests and how to apply them can be found in Supplementary Materials.

One serious issue with Ganeshan’s work is the categorical approach to transitivity she assumes compounded with a lack of statistical analysis of the data. She claims that of the 10 transitivity parameters, only agentivity, volitionality and affectedness of the object are relevant for the clitic case alternation. However, she bases this claim on the fact that both accusative and dative marking can appear in negative and non-negative sentences, for example, and therefore she concludes that negation cannot be relevant for clitic case in this construction (and the same argument is used to discard the rest of the parameters). From a probabilistic approach to linguistic variation, this conclusion is unfounded. As I said above, this type of alternation is not one of grammatical vs. ungrammatical cases but rather it is of a probabilistic nature. The question is not whether, for example, negation in itself determines the case of the clitic but whether negation favors, in a probabilistic fashion, either of the two cases. By the same token, it is not true that all sentences with an agentive subject must have a dative clitic. What might be true is that sentences with agentive subjects significantly *favor* dative marking. This, however, cannot be established from a qualitative analysis of the data.

The results from previous research make testable predictions that can be tested on new data, which will be the basis of the research questions the present work seeks to answer. Before presenting the research questions in the present study, however, we must first introduce the new predictor variable that I will add to the set of possible predictors of the clitic case alternation we are concerned with.

## Measuring the Association Strength Between the Clitic and the Verb

It goes without saying that words tend to co-occur more often with some words than with others. For example, the word *course* in English is very likely to co-occur with *of* to form the expression *of course.* Likewise, the verb *make* is more likely to appear with *sure* than with *peace*. The tendency of some words to co-occur together has been known since at least the 1930s when John Rupert Firth coined the term *collocation* (Evert, 2005). Some authors distinguish between collocation and co-occurrence, the former being a combination of words whose meaning cannot be derived compositionally, and the latter being just the observable co-occurrence frequency information as an indication of statistical association (Sinclair, 1991; Evert, 2005). For the present purposes, I use the term collocation and co-occurrence interchangeably, as a generic term to indicate two words that are more likely to appear together when compared to other words.

Since the term collocation was first coined, a lot of research has been devoted to investigating the best way to quantify and represent the notion of collocation or co-occurrence. These measures are generally known as association measures, which compute an association score for each word pair in a corpus (Evert, 2005). There are dozens of proposed association measures each with their own weaknesses and strengths as they tend to focus on different properties of collocability (Evert, 2005; Gablasova et al., 2017; see Pecina, 2010 for a study of over 80 different measures). Some measures are unidirectional or asymmetric, meaning that they distinguish between the association between word A and word B and between word B and word A. Other measures are bidirectional or symmetrical as they measure the overall association of the two words together.

In order to calculate any association measure, a number of different frequency values from a corpus are needed. These are represented in Table 2 with a specific example of the expression *of course* to make things easier to interpret.

**TABLE 2:** Co-ocurrence frequency table for calculation of association measures.

	course	Not *course*	
of	O_11_	O_12_	R_1_
Not *of*	O_21_	O_22_	R_2_
	C_1_	C_2_	N

The simplest unidirectional measure is the conditional probability of word A given word B and vice versa. These measures are also called *Attraction* and *Reliance* (Schmid, 2000) and their equations are given in (3a-b). With our example of the expression *of course*, attraction measures the strength between *course* and *of.* Reliance, on the other hand, measures the association strength between *of* and *course.* One can predict that attraction will be higher than reliance in this case because *of*, being a preposition, can be followed by potentially any noun; it is less selective. Another way of thinking about this is to say that it is easier to predict *of* given *course* than to predict *course* given *of.* This is in fact what we find using the British National Corpus; the attraction score for this phrase is 0.63 while reliance is only 0.01.

Most association measures are, however, bidirectional. The best known of these are the Mutual Information score (MI), T-score and Log-Likelihood (Gries and Ellis, 2015). These measures test the null hypothesis that the co-occurrence frequency of two words (or more) is statistically higher than chance. In other words, they assume that all words are equally likely to occur together. This assumption is clearly violated in natural language as there are semantic and syntactic constraints that limit the possibilities of two words co-occurring. To solve this shortcoming Rychlý (2008) proposed a new measure called Log Dice, whose equation is shown in (4). This measure takes the harmonic mean of two proportions that express the tendency of two words to co-occur relative to the frequency of the two words in the corpus. The advantage of this measure is that it is standardized with a maximum value of 14 and does not take into account the size of the corpus, making it possible to compare co-occurrences across corpora. A negative value indicates no statistical significance of the co-occurrence. The score measures the exclusivity of the co-occurrence, but it is not sensitive to rare combinations unlike the MI score (Evert, 2005; Gablasova et al., 2017; Messaoudi, 2019). Some say the Log Dice measures typicality of co-occurrence more than exclusivity (David, 2021). The Log Dice of the expression *of course* is 8.31. As a way of comparison, *coca cola* and *zig zag* have both a Log Dice of over 13 (Gablasova et al., 2017).

One important aspect of association measures is the extent to which they can represent cognitive aspects of language use. Researchers have probed into the cognitive validity of association measures by using them to successfully predict human behavior in a variety of linguistic tasks and phenomena (Wiechmann, 2008; Ellis and Ferreira-Junior, 2009; Gries, 2013; Gablasova et al., 2017; Levshina, 2018; Schneider, 2020; Li et al., 2021, among others).

With respect to applying association measures to the analysis of morphosyntactic variation, Gries and Stefanowitsch (2004) introduce what they call *distinctive collexeme analysis*, which is an application of association measures to the study of alternating constructions such as *passive* vs. *active* sentences, the ditransitive construction (*give a book to Mary* vs. *give Mary a book*) and *will* vs. *going-to* future. They demonstrate that association measures can be used not only for quantifying the association between two lexical words but also between morphosyntactic elements and the constructions they occur in. More recently, researchers in this area have started to apply these association measures as predictors in statistical models. Levshina (2018) shows that the alternation between *help + infinitive* and *help + to + infinitive* in English can be predicted from the Attraction and Reliance scores between *help* and the infinitive verb in seven varieties of English. In a similar vein, in a study of *that*-omission in English with both native and L2 speakers, Gries (2021) demonstrates that the unidirectional association between the main verb and *that* is one of the predictors of *that-*omission.

A result that has become clear out of the research on association measures is that the choice of the association measure may impact the results and there is no one single association score that has been shown to outperform all others across tasks and/or phenomena. Thus, it is recommended that a number of association measures should be assessed before choosing one specific measure for a particular study. In the present article, I compared Attraction, Reliance and Log Dice in terms of their predictive power of clitic case before selecting Log Dice as the association measure to be used. The choice of these measures was based on three main factors. First, I wanted to assess the difference between a bidirectional and a unidirectional measure since unidirectional measures may be more sensitive than bidirectional ones as they quantify two different types of associations as illustrated above. Second, there were many examples in the dataset with very low frequencies and zeroes, so it was important to use measures that were not biased toward low frequency combinations as, for example, the MI score. Third, I was interested in studying the effect of measures whose mathematical form did not assume the null hypothesis of free association of all words since this is not an appropriate model of language, despite the fact that they may still be good predictors.

$$3.a.\text{Attraction}=\frac{{\text{O}}_{11}}{{\text{O}}_{11}+{\text{O}}_{21}}\;b.\text{Reliance}=\frac{{\text{O}}_{11}}{{\text{O}}_{11}+{\text{O}}_{12}}$$

$$4.\mathrm{Log}\,\mathrm{Dice}=14+{\text{Log}}_{2}\,\left(\frac{2\,{\text{O}}_{11}}{{\text{R}}_{1}+{\text{C}}_{1}}\right)$$

Having presented the previous research on clitic case alternation with reverse psychological predicates and the association measures to be tested as new predictors, I will now present the research questions, hypotheses and predictions of the current study.

## Research Questions, Hypotheses And Predictions

The findings presented from the previous literature allow us to ask specific research questions and propose clear hypotheses that we can test with the help of new data and a statistical model. In this section, I present the motivation for this study that serves as the backbone of the article.

**Research question 1:**

– Is transitivity, computed as a single composite score of the whole clause containing the clitic, predictive of clitic case?

**Research question 2:**

– Is there a North-South cline in American Spanish in the alternation of the clitic case with r-psych verbs?

**Research question 3:**

– Which parameter is more important in the clitic case alternation: animacy (Miglio et al., 2013) or agentivity of the subject (Ganeshan, 2015)?

**Research question 4:**

– Are there signs of lexicalization of the *clitic+verb* combination such that certain verbs are more likely to appear with one of the two clitics?

With research question (RQ) 1, I seek to determine whether transitivity as a property of the whole clause is really predictive of clitic case. Since most studies have selectively chosen a specific subset of the parameters, it remains to be demonstrated that transitivity as a whole is what drives the clitic case alternation with r-psych verbs. To reiterate, according to Hopper and Thompson’s (1980) proposal individual parameters do not determine the transitivity value of a clause. By selecting and focusing on individual parameters, one runs the risk of drawing generalizations that may not be an appropriate representation of the phenomenon. The null hypothesis is that transitivity is not predictive of clitic case. However, the prediction based on the previous literature is that transitivity will be a significant factor such that the dative clitic will be associated with lower levels of transitivity.

RQ-2 seeks to test the claim in Miglio et al.’s (2013) work that there appears to be a North-South cline of accusative use, where the further South one goes, the higher the probability of the accusative clitic. Recall that their finding seems to be better interpreted as a difference between Mexico and the rest of Latin American (as Figure 1 suggests) rather than a gradual cline. Thus, the prediction for RQ2 is that there will be a difference in the use of clitic case between Mexico and the rest of the Latin American varieties, with Mexico generally preferring the dative clitic over the accusative.

RQ-3 seeks to determine which of the two features of the subject, animacy or agentivity, are more important in the alternation. Ganeshan (2015) claims that animacy is really a proxy for agentivity but agentivity is the parameter that is relevant. However, neither Miglio et al. (2013) nor Ganeshan (2015) tested both parameters simultaneously. No particular prediction is possible with this RQ as we are seeking to determine which of these two options turns out to be supported by the data.

RQ-4 concerns the issue of whether some *clitic+verb* combinations may be more likely than others such that their association strength will be predictive of the clitic. My hypothesis is that the association strength between the verb and the clitic will be distinctive, thus the prediction is that different verbs will show a preference for one of the two clitics.

## Materials and Methods

In this section, I present the methodology for data extraction and annotation, I describe how the two continuous variables were calculated and conclude with a description of the statistical model. The statistical analysis was conducted in R 4.0.3 (R Core Team, 2020).

### Data Extraction

The data was extracted from Corpus del Español (Davies, 2016), a corpus with nearly 2 billion words that is annotated for parts of speech. The corpus contains data from 21 Spanish-speaking countries including the United States. Data extraction was carried out by the author through the web interface of the corpus. Ganeshan (2015) provides a list of 40 r-psych verbs that allow the clitic-case alternation so each of these verbs was searched in the corpus. The search consisted of the lemma of the verb preceded by either the accusative (in both numbers and genders) or the dative clitic (both singular and plural) with a minimum frequency of three. For each verb a random sample of 100 sentences was obtained. When the corpus returned fewer than 100 hits, all of them were kept. The final list of verbs contains 37 predicates because three of them returned no hits (*desanimar* “discourage”and *desconsolar* “distress”) or very few (*pasmar* “astonish”). The list of verbs is provided in Supplementary Materials.

The data from Spain and the United States was removed because of *leismo* with respect to Spain and because in the United States there are speakers of other regions as well as second language speakers and this would introduce extra noise in the statistical model. After removal of duplicates and false positives, the final dataset contains a total of 4017 observations, with a relative frequency of 0.54 and 0.46 for the accusative and the dative clitic, respectively.

The data was annotated by a trained research assistant for the transitivity parameters and three additional variables, namely TENSE, SUBJTYPE and PERSON. The variable TENSE is binary with values perfective and non-perfective^4^. This variable then distinguishes between perfective tenses (preterite)^5^ and non-perfective tenses (present, imperfect, future, conditional and past subjunctive). The variable SUBJTYPE refers to whether the subject of the r-psych verb was a lexical NP or a clause. PERSON is also a binary predictor whose values can be 3rd or non-3rd person.

### The Continuous Predictors

The main predictor variables in this article are the association measure and the Transitivity Index. Since the dataset contains observations from 19 Spanish-speaking countries, the association measures were calculated by country, so for each *clitic+verb* combination there are 19 values for each association measure. This allows us to examine regional variation in more detail. The frequencies for the calculation of the association measures were taken from Corpus del Español, Web/Dialects version, which is the same corpus the data was extracted from.

The Transitivity Index (Guajardo, 2021) was calculated by training 1,000 large random forests of 3,000 trees with the ranger package (Wright and Ziegler, 2017) on a subset of the data (22%) containing all and only the transitivity parameters coded for low and high. This subset of the data was only used for the calculation of the Transitivity Index and then put aside for the rest of the analysis. The permutation variable importance was calculated for each random forest and the final variable importance is the average of the 1,000 variable importance scores. This results in each parameter having a mean importance score indicating how predictive of the outcome they are. The more predictive a parameter is, the higher the variable importance score it will receive. Subsequently, each high value of the transitivity parameters is replaced with its importance score and each low value gets zero. The final step consists of adding up all the parameter scores per data point in the dataset such that each example of *clitic+verb* receives a total transitivity score. This final score, the Transitivity Index, can then be used as a predictor in any statistical model. Table 3 shows the final parameter weights as a result of this procedure.

**TABLE 3:** Mean weights of the transitivity parameters.

Parameter	Mean	Parameter	Parameter	Mean	Parameter
Punctuality	0.048260	Affectedness	0.002202	Agency-subj	–0.000012
Individuation_*O*__*bj*_	0.032610	Affirmation	0.001525	Mode	–0.000192
Individuation_*S*__*ubj*_	0.017821	Kinesis	0.000007	Aspect	–0.00026

Both continuous variables had to be transformed because they did not have a linear relationship with the dependent variable. The association measures were rank-transformed and then normalized from 0 to 14 to resemble the original scale. The Transitivity Index was transformed using the negative logarithm of the cosine. The negative logarithm was used to change the sign and keep the directionality of the index as the original so that an increase in the index meant an increase in transitivity. This score was then normalized between 0 and 1 as the original scale.

### Statistical Analysis

The first step in the analysis was to assess which of the three association measures best predicted the case of the clitic. To do this, I trained a random forest with 3,000 trees with the ranger package (Wright and Ziegler, 2017) and calculated the permutation variable importance. Random forest was used in this step because the three association measures are correlated and random forests can handle correlated variables (Tomashek et al., 2018). The second step consisted of fitting a mixed-effects logistic regression model with the lme4 package (Bates et al., 2015) with WEBSITE as random intercept. The website address of each observation indicates the source of each data point. COUNTRY was not included as a random effect because I was interested in testing whether there was a North-South cline. Thus, I created a new variable called VARIETY by grouping the 19 countries into five regions or varieties as in Miglio et al. (2013) and this variable was included in the model as a fixed effect. The countries comprising each variety are shown in Table 4. In addition, because Log Dice was calculated by country and verb, VERB was not included as a random effect as the potential differences among the verbs are already included in the Log Dice score.

**TABLE 4:** The composition of the predictor variable VARIETY

Variety	Country	Variety	Country
Mexico	Mexico	South Am	Bolivia
Caribbean	Cuba		Colombia
	Dominican Republic		Ecuador
	Puerto Rico		Peru
Central Am	Costa Rica		Venezuela
	El Salvador	Southern Cone	Argentina
	Guatemala		Chile
	Honduras		Paraguay
	Nicaragua		Uruguay
	Panama		

Continuous variables were centered on the mean and scaled. Binary factors were entered into the model with sum contrasts and VARIETY used Helmert contrasts to compare Mexico with the rest of the regions. Model selection was performed by means of log-likelihood ratio tests of nested models.

## Results

The random forest selected Log Dice as the most predictive among the three association measures, so this was the variable used for the logistic regression model. In Table 5, I present the untransformed means for Log Dice and the Transitivity Index for the accusative and the dative clitics across all countries. We can see that the dative clitic has a higher mean Log Dice (2.62 vs. 1.30) but a lower mean Transitivity (0.50 vs. 0.57) compared to the accusative clitic. We can also see that the differences are much larger in the Log Dice than in the Transitivity Index, with the Log Dice of the dative clitic being twice as high as the accusative. In addition, it seems that the dative clitic shows no preference in terms of transitivity as its mean is 0.50, indicating that it is likely to appear in either a high or a low transitivity context.

**TABLE 5:** Overall Log Dice and transitivity index means across all countries and verbs.

	Accusative	Dative
Log Dice	1.30	2.62
Transitivity Index	0.57	0.50

The mixed-effects logistic regression model (*C*-index = 0.87; *R*^2^ marginal = 0.41, *R*^2^ conditional = 0.47)^6^ contains SUBJTYPE, PERSON and TENSE as single terms and the interactions LOG DICE^∗^VARIETY and TRANSITIVITY^∗^VARIETY. In addition, it contained WEBSITE as a random intercept.

There were significant main effects for LOG DICE (χ^2^ = 424.51, *p* < 0.001), TRANSITIVITY (χ^2^ = 20.64, *p* < 0.001), VARIETY (χ^2^ = 91.01, *p* < 0.001), TENSE (χ^2^ = 12.69, *p* < 0.001), SUBJTYPE (χ^2^ = 98.84, *p* < 0.001), and PERSON (χ^2^ = 77.55, *p* < 0.001). The interaction LOG DICE^∗^VARIETY was found to be significant (χ^2^ = 182.41, *p* < 0.001) as well as the interaction TRANSITIVITY^∗^VARIETY (χ^2^ = 16.13, *p* < 0.005). No other significant interactions were found. I will illustrate the results by means of marginal effects as they are a very reader-friendly way to interpret the results of the model^7^. The marginal effects were calculated with the ggeffects package (Lüdecke, 2018).

**FIGURE 2:**
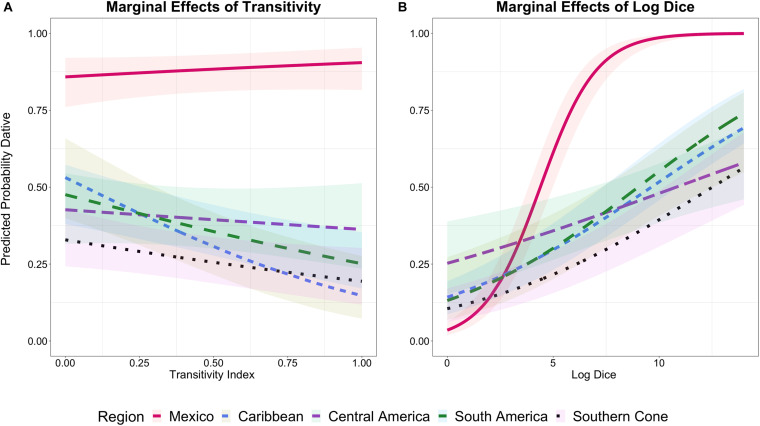
Marginal effects of Log Dice and Transitivity Index on clitic case. The *y*-axis represents the predicted probability of the dative clitic. In **(A)**, the *x*-axis represents the Transitivity Index between 0-1 and in **(B)** the Log Dice between 0 and 14.

Figure 2 shows the marginal effects of Log Dice and the Transitivity Index by region. The results show a clear difference between Mexico and the rest of the regions for both predictors. The marginal effects for the Transitivity Index in Figure 2A show that, in general, Mexico has a nearly categorical preference for the dative clitic regardless of transitivity, and it significantly favors the dative clitic as transitivity increases compared to the other four regions combined (β_*Mexico*_ = 0.43, CI: [0.16, 0.69], *p* < 0.05). A *post-hoc* pairwise Tukey test comparison shows no difference between the Caribbean, Central America, South America and Southern Cone regions. The pairwise comparison also shows that the differences obtained in the model between Mexico and the four regions combined is driven by a statistically significant difference between Mexico-Caribbean (*p* < 0.05) and Mexico-South America (*p* < 0.05). As the plot shows, in the Caribbean and South America regions the predicted probability of the dative clitic decreases with higher levels of transitivity.

In Figure 2B, Mexico shows a very strong, nearly categorical, preference for the dative clitic with Log Dice values higher than eight. This is also reflected in the positive coefficient of the model (β_*Mexico*_ = 2.40, CI: [1.91, 2.82], *p* < 0.001), which means that, compared to the other four regions combined, Mexican Spanish significantly favors the dative clitic as the Log Dice score increases. The other regions also show a preference for dative marking with higher scores of Log Dice, but the slopes of the curves are much more gradual, and they never reach a predicted probability of 1 unlike Mexico. A *post-hoc* pairwise Tukey test showed a statistically significant difference between Mexico and every one of the other four regions (*p* < 0.001) and between Central America and South America (*p* < 0.05), with South America favoring the dative clitic to a higher degree than Central America. No other significant differences were found between the regions with respect to Log Dice.

**FIGURE 3:**
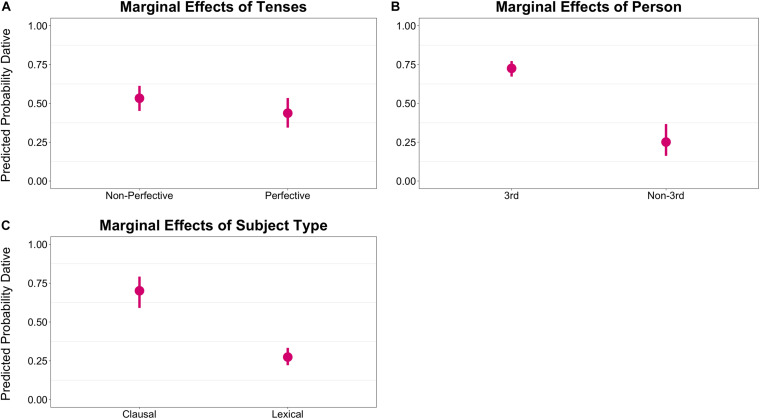
Marginal effects of the single terms on clitic case. The *y*-axis represents the predicted probability of dative clitic. **(A)** shows the marginal effects of TENSE, **(B)** shows the effects of PERSON and **(C)** SUBJTYPE.

Figure 3 shows the marginal effects of the single terms in the model. Figure 3A shows the marginal effects of TENSE, with non-perfective tenses showing a slightly higher preference for the dative clitic (β = 0.19, CI: [0.05, 0.31], *p* < 0.05). Figures 3B,C show the marginal effects of person and subject type, respectively. The results show a stark contrast between third vs. non-third person subjects (β = 1.03, CI: [0.77, 1.28], *p* < 0.001) and clausal vs. lexical NP subjects (β = 0.91, CI: [0.69, 1.09], *p* < 0.001). Both third person and clausal subjects show a strong preference for the dative clitic with a predicted probability of over 0.70 for the dative clitic compared to a predicted probability of around 0.25 for non-third persons and lexical NPs.

## Discussion

In this section, I will first summarize the main findings presented above and then assess the hypotheses and predictions based on these results. I will conclude the section with an interpretation of the findings in light of transitivity and the association strength between the clitic and the verb.

### The Main Findings

As is clear from the Results section, we found relatively large differences between Mexico and the other four regions, whereas we did not find as many differences between the four remaining regions. In fact, the only difference found in these four regions was between Central and South America with respect to Log Dice. Regional variation is more pronounced with respect to Log Dice because Mexico appears to be different from every single one of the four regions. The regions are more homogenous when it comes to transitivity, with transitivity having a very small effect or no effect depending on the region. In addition, we confirmed previous findings showing that perfective tenses and clausal subjects show a preference for dative marking as do third person subjects. With these results in mind, we will now assess the hypotheses and predictions laid out above.

### Assessing the Research Questions, Hypotheses and Predictions

As the reader may recall, the present study is guided by four research questions. Thus, in this section I will review each of the research questions with their hypotheses and predictions when applicable in light of the results reported in the previous section. I will include the research questions here as well for ease of readability.

**RQ 1:**

– Is transitivity, computed as a single composite score of the whole clause containing the clitic, predictive of clitic case?

Based on previous literature, it was predicted that higher levels of transitivity would be associated with accusative marking. The results show that this is indeed the case. We found a main effect of transitivity and it shows that as transitivity increases the odds of finding the dative clitic decrease. However, we found important regional differences, which leads us to our second research question.

**RQ 2:**

– Is there a North-South cline in the alternation of the clitic case with r-psych verbs?

Based on a re-assessment of Miglio et al. (2013)’s results, we predicted there should be significant differences between Mexico and the other four regions combined, with Mexico showing a higher preference for dative marking. We did not expect to find a gradual cline but simply two main groups: Mexico vs. the rest.

The results we obtained are mixed. On the one hand, we did find a highly significant difference between Mexico and the other four regions *combined* for Log Dice, and we also found a statistically significant difference between Mexico and the other four regions for transitivity, though this latter difference is not as large. On closer inspection, pairwise comparisons revealed that Mexico is indeed different from every single region when it comes to Log Dice but not for transitivity. The difference in transitivity reported in the model was driven by the Caribbean and South America regions but no difference in transitivity was found between Mexico-Central America and Mexico-Southern Cone. Thus, the results suggest that Mexico does indeed prefer dative marking with r-psych predicates to a greater extent than the Caribbean, Central America, South America and the Southern Cone regions but transitivity does not seem to play a very important role in clitic choice in this region. Moreover, the results do not support the existence of a gradual North-South cline. For Log Dice there appear to be two major regions as we predicted (i.e., Mexico vs. the rest) whereas for transitivity the picture is much less clear and points to non-contiguous regional differences rather than a gradual cline.

**RQ 3:**

– Which parameter is more important in the clitic case alternation: animacy (Miglio et al., 2013) or agentivity of the subject (Ganeshan, 2015)?

This question was inspired by the two previous claims in the literature where Miglio et al. (2013) found animacy of the subject to be the main predictor of clitic case whereas Ganeshan (2015) claimed it was agentivity, with animacy being simply a proxy for agentivity.

The results of the parameter weights showed that animacy received a much higher weight than agentivity (0.003 vs. – 0.00001). Hence, it seems that animacy plays a much more important role than agentivity in predicting clitic case with r-psych predicates.

**RQ 4:**

– Are there signs of lexicalization of the *clitic+verb* combination such that certain verbs are more likely to appear with one of the two clitics?

This research question was aimed at studying the extent to which the alternation in clitic case could be accounted for by transitivity or by lexical preferences of each of the verbs.

The results show a clear large effect of Log Dice showing that clitic case is highly predictable from the association strength between each verb and the clitic. In fact, Log Dice is a much stronger predictor than transitivity. Comparing a base model where all the predictors are single terms (i.e., no interactions) with a model where Log Dice has been removed results in a chi-squared of 424.51 and *R*^2^-marginal of 0.12. The same comparison but with Transitivity removed produces a chi-squared of 20.64 and a *R*^2^-marginal of 0.26. Thus, removing Log Dice from the model decreases its predictive power by a larger magnitude than transitivity.

### Final Remarks

The fact that Log Dice turned out to be more predictive of clitic case than transitivity suggests that the alternation is more dependent on each particular verb than previously thought. Another way to interpret this result is that the dative clitic is the default or unmarked form in this construction and hence shows a higher degree of association with all verbs participating in the construction. Recall that the class of verbs that allows the alternation is just a subclass of the r-psych-verbs in Spanish. The other class of psych-verbs that does not follow the usual nominative-accusative marking only allows dative marking of the experiencer such as *gustar* “to like,*” encantar* “to love” and *faltar* “to lack.” Although this class of predicates is fewer in type (Vázquez Rosas, 2006; Fábregas and Marín, 2015), their token frequency is very high and can therefore exert an influence on the other less frequent predicates that allow for both cases of the clitic (Bybee and Thompson, 1997; Bybee, 2003, 2007). As a way of example, the three predicates mentioned above (*gustar* “to like,*” encantar* “to love” and *faltar* “to lack”) have an average frequency of 261.58 per-million words in the corpus. In contrast, the three verbs that have the highest Log Dice score in our dataset are *molestar* “to bother,*” sorprender* “to surprise” and *apasionar* “to be passionate about,” which have an average per-million frequency of 48.15. Thus, because the nominative-dative pattern with psych-verbs has such high token frequency, it is likely to attract members of the neighboring class and render dative marking the default option with this type of predicate.

Note, in addition, that the effects of Log Dice and transitivity appear to be independent of each other as we found no significant interaction between the two. This, in turn, supports the finding that the effect of transitivity is rather small. If the dative clitic were strongly associated with lower levels of transitivity, then we should also have found a negative correlation between Log Dice and transitivity, because higher levels of transitivity should correspond to *less* dative marking whereas higher values of Log Dice correspond to *more* dative marking. The lack of this correlation (*r*_*s*_ = −0.10) seems to suggest that the dative clitic is simply the unmarked form in this construction and the accusative clitic potentially carries more semantic weight. However, it is up to the speaker whether they choose to signal this difference via a change in the case of the clitic and this is likely why the effect of transitivity is relatively small. That is, the association of the clitic with the verb seems to override the potential effect of transitivity.

The present results are partially in line with Ganeshan’s claim that accusative marking is unspecified for agentivity and entails affectedness of the object, while dative case entails a weakening or lack of agentivity and is unspecified for affectedness of the object. That is, the Transitivity Index indicates that dative marking is unmarked for transitivity with a mean transitivity of 0.50 and this is similar to Ganeshan’s idea that the dative signals lack of agentivity and affectedness. In my analysis then, the dative clitic is simply unspecified for transitivity whereas the accusative clitic signals higher transitivity. The accusative clitic, however, does not seem to signal affectedness as Ganeshan suggests, since the mean for Affectedness for both clitics is 0.002. However, recall that Table 3 shows that Punctuality, Individuation of the object and Individuation of the subject are the three most important parameters. If we look at the means of these parameters, we can see that the accusative has a higher mean for Punctuality (0.024 vs. 0.015) and Individuation_*Subj*_ (0.007 vs. 0.003) but a lower mean for Individuation_*Obj*_ (0.024 vs. 0.032). Thus, the accusative clitic signals a punctual event with a highly individuated subject whereas the dative clitic is neutral with regards to general transitivity but is more likely with individuated objects.

A question raised by one reviewer is whether there are other changes in Mexican Spanish that could be related to the results of the present work. One possible development that can be established along these lines is the use of the dative clitic as an intensifier or verb modifier in Mexican Spanish. This is a relatively well-known and highly productive phenomenon in this variety where the dative clitic appears with either a transitive or intransitive verb to express an intensive meaning as in (5) (Torres Cacoullos, 2002; Navarro-Ibarra, 2007; Navarro and Espinal, 2012). Crucially, in this innovative use, the dative clitic can alternate with the accusative. In other words, as a result of this productive phenomenon, the dative clitic shows up in contexts where one would only expect the accusative clitic in standard Spanish. In Mexican Spanish, however, this is yet another context of clitic case variation. Thus, it may be that the dative clitic in Mexican Spanish is expanding its contexts of use to formerly exclusively accusative contexts. As a result of the high and growing productivity of this construction, the dative clitic is increasing its token and type frequency, which, as is well-known, can have profound consequences in neighboring constructions where the dative clitic also alternates with the accusative clitic.

5. a. Trae unos “Raleigh.” ¡Córrele!
  Bring some Raleigh run-LE
  “Bring some ‘Raleighs.’ Go on, run!”
  (Torres Cacoullos, 2002, p. 285)
b. Ya le sabe
  already LE knows
  “She knows how to do it, she has figured it out”
  (Torres Cacoullos, 2002, p. 287)

It should also be noted that the results about the strong associative relationship between the verb and the clitic cast doubt on Vázquez Rosas’ claim that “the majority of the verbs have not lexicalized the accusative or the dative construction […]” (Vázquez Rosas, 2006, p. 108). It seems that, on the contrary, verbs do generally default to dative marking unless the stimulus is not clausal or third person or the speaker chooses to highlight higher transitivity.

An interesting parallel with the results obtained is the discussion in Pineda (2020) about a variety of agentive verbs in Catalan and Spanish that also allow clitic case alternation to different degrees^8^. She argues that the alternation is a reflex of different stages in the grammaticalization cline from dative to accusative marking resulting in a complete transitivisation of the structure at the completion of the process. Similar to our results, the accusative pattern results in the object being interpreted as a patient, i.e., signaling higher transitivity. It will be interesting to see whether similar grammaticalisation stages can be identified with r-psych verbs in future work.

An important take-away message from the present study is that clitic case alternation is optional in a probabilistic sense. There is individual, regional and contextual variability and neither of the two case markings can render a sentence grammatical or ungrammatical. It is simply a matter of what is *more likely* to be said under different conditions. This state of affairs poses challenges for formal analyses that present this phenomenon as dichotomous with very distinct and mutually exclusive interpretations for each clitic (cf. Parodi and Luján, 2000; Ackerman and Moore, 2001; Fábregas et al., 2017; Fábregas and Marín, 2020).

To sum up, the main contribution of the present article is the finding that there is a very strong lexical association between the clitic and the verb with r-psych predicates that allow for both accusative and dative marking of the clitic. Crucially, the results indicate that the *clitic+verb* association is much more predictive of clitic case than transitivity, which I have interpreted as suggesting that the dative clitic appears to be the default form in this construction and the accusative clitic carries an extra layer of meaning that the speaker may choose to exploit.

Methodologically, I have shown the advantage and practicality of operationalizing transitivity as a continuous measure by incorporating all the parameters in one single score, thus allowing us to study the effect of transitivity as a global property of the clause without an arbitrary choice of single parameters.

## Data Availability Statement

The datasets presented in this study can be found in online repositories. The names of the repository/repositories and accession number(s) can be found below: https://rb.gy/ycjsnc.

## Author Contributions

The author confirms being the sole contributor of this work and has approved it for publication.

## Conflict of Interest

The author declares that the research was conducted in the absence of any commercial or financial relationships that could be construed as a potential conflict of interest.

## Footnotes

1 The country and number below each example refer to the country tag of the sentence according to the corpus and the ID number in the dataset. The dataset and R-code analyzed for this study can be found on the Open Science Framework website on shorturl.at/BRV49.

2 The authors do not mention which countries are included in each region.

3 With respect to Latin America, Parodi et al. (2012) show that *leísmo* has been completely absent in this region since the 20th century. The exception to this finding is in cases of language contact such as the rural areas of the Andean region, Paraguay and Ecuador (see also Demello, 2002 for similar conclusions).

4 Although perfective vs. imperfective refers more to an aspectual distinction than a temporal one, I keep the name TENSE for this variable because ASPECT is one of the transitivity parameters.

5 There were no examples of perfect in the dataset.

6 The coefficient of determination *R*^2^ ranges from 0 to 1 and represents the proportion of variance of the dependent variable that is explained by the model. The marginal *R*^2^ refers to the variance explained by the fixed effects only while the conditional *R*^2^ is the variance explained by both the fixed and the random effects. The *R*^2^ reported here is the one developed by Nakagawa and Schielzeth (2013) available in the piecewiseSEM package in R.

7 A complete table with coefficient estimates, standard errors, confidence intervals and *p*-values can be found in Supplementary Materials.

8 I thank an reviewer for referring me to this article.
