# Supplementary material for: Co-occurrence Strength and Transitivity Effects on Spanish Clitic Case Variation With Reverse-Psychological Predicates
Source: Front Psychol. 2021 Jul 19;12:712959. doi: 10.3389/fpsyg.2021.712959 (PMC8330882; doi:10.3389/fpsyg.2021.712959)
Supplement: Supplementary file 1 [file Data_Sheet_1.PDF]

Supplementary Material

The following is the list of the reverse psychological predicates used in this study. The list is taken from Ganeshan (2015).

abrumar 'to overwhelm'
a*burrir* 'to bore'
alegrar 'to make happy'
angustiar 'to distress'
apasionar 'to make passionate'
apenar 'to sadden'
asombrar 'to amaze'
asustar 'to frighten'
atemorizar 'to frighten'
aterrar 'to terrify'
aterrorizar 'to terrorize'
atormentar 'to torment'
avergonzar 'to shame'
cansar 'to tire'
complacer 'to please'
decepcionar 'to disappoint'
deanimar 'to discourage'
desconsolar 'to distress'
desesperar 'to exasperate'
disgustar 'to disgust'
divertir 'to amuse'
emocionar 'to move/be touching'
entristecer 'to sadden'
entusiasmar 'to encourage'
espantar 'to scare'
estorbar 'to bother'
exasperar 'to exasperate'
fascinar 'to fascinate'
fastidiar 'to annoy'
impresionar 'to impress'
incomodar 'to inconvenience'
inquietar 'to unsettle
intranquilizar 'to worry'
irritar 'to irritate'
molestar 'to bother'
ofender 'to offend'
pasmar 'to astonish'
preocupar 'to worry'
sorprender 'to surprise'
tranquilizar 'to calm down'

Diagnostics for Action, Volitionality, Agentivity and Affectedness from Ganeshan (2015).

- **Action:** *y X lo hace/ hizo + modifier* 'and X does/did it + modifier'
  Modifiers: *apasionadamente* 'passionately', *apuradamente* 'hurriedly', *frenéticamente* 'frenetically.

Explanation: If the sentence is compatible with these adverbs, then the verb describes an action.

Example: El niño rompió el vaso y lo hizo *frenéticamente*
'The child broke the glass and he did it frenetically'
(Ganeshan 2015:128)

- **Volitionality:** modify the clause with adverbs such as *deliberadamente* 'deliberately', *a propósito* 'on purpose', *por propia voluntad* 'by his/her own will', *de mala gana* 'reluctantly', *con pocas ganas* 'with little interest'.

Explanation: If the sentence is incompatible with these expressions,, then the predicate is volitional. If the sentence can be modified by *accidentalmente* 'accidentally' then the predicate is non-volitional.

Example: #Juan asesino a Maria accidentalmente
'Juan (intentionally) killed Maria by accident'

(Ganeshan 2015:123)

- **Agentivity:** a predicate that is neither volitional nor an action is deemed non-agentive.
- **Affectedness:**
    - *quedar + adjectival participle + por X tiempo* ''to remain + adjectival participle + for X time'.
    - φ, and still ψ entailment

Explanation: If the sentence is compatible with either of these constructions, then the object is affected.

Example: Juan rompió el vaso (y el vaso quedo roto por años).
'Juan broke the glass and the glass remained broken for years'

Juan rompió el vaso y el vaso todavía está roto.
'Juan broke the glass and the glass is still broken'

(Ganeshan 2015: 133)

Table 1. Coefficient estimates, standard errors, interval coefficients and *p*-values of the mixed-effects logistic regression model.

	Estimate	Std.Error	CI-L	CI-U	z-value	p-value	
(Intercept)	-0.061	0.173	-0.399	0.295	-0.352	0.724	
LogDice	1.153	0.075	0.983	1.250	15.363	<0.001	***
Variety- Mexico	2.513	0.259	2.006	2.999	9.688	<0.001	***
Variety-Caribbean	0.091	0.167	-0.259	0.412	0.545	0.586	
Variety-Central America	0.303	0.143	0.017	0.574	2.121	0.034	*
Variety-South America	0.522	0.126	0.286	0.739	4.145	<0.001	***
SubjType: Clausal	0.913	0.106	0.690	1.092	8.577	<0.001	***
Person: 3rd	1.033	0.132	0.766	1.280	7.802	<0.001	***
Transitivity	-0.207	0.053	-0.310	-0.101	-3.939	<0.001	***
Tense: Non-Perfective	0.193	0.065	0.051	0.315	2.986	0.003	**
LogDice*Variety-Mexico	2.381	0.242	1.908	2.818	9.858	<0.001	***
LogDice*Variety-Caribbean	0.105	0.176	-0.207	0.438	0.597	0.551	
LogDice*Variety-Central America	-0.365	0.133	-0.621	-0.100	-2.745	0.006	**
LogDice*Variety-South America	0.155	0.135	-0.098	0.390	1.146	0.252	
Transitivity*Variety-Mexico	0.429	0.141	0.162	0.695	3.038	0.002	**
Transitivity*Variety-Caribbean	-0.371	0.156	-0.667	-0.064	-2.377	0.017	*
Transitivity*Variety-Central America	0.178	0.129	-0.094	0.413	1.377	0.169	
Transitivity*Variety-South America	-0.086	0.127	-0.324	0.125	-0.682	0.495	
